# Molecular Signatures of Maladaptive Plasticity in the Amygdala in a Rat Model of Chronic Neuropathic Pain

**DOI:** 10.3390/cells15090775

**Published:** 2026-04-25

**Authors:** Peyton Presto, Julian Cardenas, Christian Bustamante, Brent R. Kisby, Guangchen Ji, Olga Ponomareva, Volker Neugebauer, Igor Ponomarev

**Affiliations:** 1 Department of Pharmacology and Neuroscience, Texas Tech University Health Sciences Center, 3601 4th Street, Lubbock, TX 79430, USA; peyton.presto@ttuhsc.edu (P.P.); brent.kisby@ttuhsc.edu (B.R.K.);; 2 Center of Excellence for Translational Neuroscience and Therapeutics, Texas Tech University Health Sciences Center, 3601 4th Street, Lubbock, TX 79430, USA; 3 Garrison Institute on Aging, Texas Tech University Health Sciences Center, 3601 4th Street, Lubbock, TX 79430, USA

**Keywords:** pain, amygdala, transcriptomics, bioinformatics, neuroimmune, myelination, neuroplasticity

## Abstract

Chronic pain, a complex multidimensional disorder, remains a major healthcare issue and a therapeutic challenge. Neuropathic pain is a chronic pain condition that results from damage or dysfunction in the nervous system. While mechanisms of neuropathic pain at the peripheral and spinal cord level have been extensively studied, pain mechanisms in the brain remain underexplored. The amygdala, a limbic brain region, has emerged as a critical brain area for the emotional–affective dimension of pain and pain modulation. Amygdala neuroplasticity has been associated with pain states, but the exact molecular and cellular mechanisms underlying these states and the transition from acute to chronic pain are not well understood. Here, we used the spinal nerve ligation (SNL) model of neuropathic pain in male rats to investigate changes in gene expression in the amygdala at the chronic pain stage using RNA sequencing (RNA-Seq). Two amygdala nuclei, the basolateral (BLA) and central (CeA), were investigated in a hemisphere-dependent manner. We used an integrative approach that focuses on functional significance and cell-type specificity of differentially expressed genes (DEGs) to nominate mechanistic targets for central regulation of chronic pain. Our integrative transcriptomic and bioinformatic analyses identified individual genes (e.g., *Cxcl10*, *Cxcl12*, *Mbp*, *Plp1*, *Mag*, *Mog*, *Slc17a6*, *Gad1*, and *Sst*), molecular pathways (e.g., cytokine-mediated signaling pathway), biological processes (e.g., myelination, synaptic transmission), and specific cell types (e.g., oligodendrocytes, glutamatergic, and GABAergic neurons) affected by chronic pain. Our results also provide some evidence for the emerging concept of hemispheric lateralization of pain processing in the amygdala. Overall, our study proposes oligodendrocyte dysfunction in the amygdala, neuroimmune signaling in the CeA, and glutamatergic neurotransmission in the BLA as key processes and potential therapeutic targets for the management of chronic neuropathic pain.

## 1. Introduction

The International Association for the Study of Pain (IASP) defines pain as “an unpleasant sensory and emotional experience associated with, or resembling that associated with, actual or potential tissue damage” [1]. Acute pain serves as a protective warning of potential danger and plays a critical role in the body’s healing response. In contrast, chronic pain is a disorder that impairs functioning and quality of life, necessitating effective treatment or management. Chronic pain, such as neuropathic pain, is difficult to treat, partly due to its highly complex nature involving brain processes that are still not well understood. The estimation of the incidence and prevalence of neuropathic pain has been difficult because of the lack of simple diagnostic criteria for large epidemiological surveys in the general population. The prevalence of chronic pain with neuropathic characteristics has been estimated to be in the range of 7–10% [2]. Drug development and drug repurposing require understanding the mechanisms underlying maladaptive processes at every level in the nervous system that are associated with the transition from acute to chronic (chronification of) pain.

The amygdala, a limbic structure located in the medial temporal lobe, has emerged as a critical brain area for the emotional–affective dimension of pain and pain modulation based on preclinical [3,4] and clinical [5,6,7] evidence. The amygdala comprises anatomically and functionally distinct nuclei and subdivisions. The regions of the amygdala that are relevant for sensory- and pain-related processing are the lateral/basolateral complex (LA/BLA), the intercalated cell (ITC) mass, and the central nucleus (CeA). The CeA is the major amygdala output center and contains efferent projections to the brainstem, hypothalamus, and basal forebrain regions [8,9,10]. Changes in amygdala neuronal activity have been observed in pain models, and neuroplasticity within the amygdala has been linked to pain-like behaviors. Pain-related neuroplastic changes lead to hyperexcitability in amygdala output neurons, driving pain-like behaviors in both acute [11,12] and chronic [13,14] pain models. Despite recent advances in dissecting the brain neurocircuitry of pain-like behaviors, specific cellular and molecular mechanisms underlying the chronification of pain are not well understood.

Gene expression (transcriptomic) analysis has been widely used for gaining insight into molecular mechanisms of brain disorders and for the discovery of novel molecular targets for medication development. Transcriptomic profiling in the periphery and spinal cord of animal pain models has revealed regulation of many genes implicated in biological processes related to neuronal functions and the immune response [15,16], though pain-related gene expression profiles within the brain are understudied. A recent study of the whole amygdala transcriptome at the acute stage (6 days) of a mouse model of neuropathic pain [17] reported several individual genes and biological functional groups affected by the induction of pain at this stage. However, important knowledge gaps remain, including the genes involved in the chronic pain condition; the role of specific cell types in different amygdala subregions, including non-neuronal cell types such as microglia and oligodendrocytes; and the potential hemispheric lateralization of the amygdala transcriptome. There is good evidence to suggest differential roles of the right and left amygdala in the regulation of pain-like behaviors, and, potentially, chronification of pain. The regulation of pain is lateralized in rodents and humans in ways that cannot be explained solely by the anatomic features of ascending sensory or descending modulatory pathways. Findings suggest a right-lateralized amplification of pain processing by neuropeptides in the CeA and parabrachial nucleus, and this asymmetry may result from lateralized circuit organization [18,19,20]. However, the molecular signatures of pain-related amygdala plasticity and the hemispheric lateralization remain to be determined.

The goal of this study was to identify molecular and cellular factors involved in pain-related neuroplasticity. Here, we characterized the transcriptional profiles of both the right and the left CeA and BLA of male rats at the chronic stage of neuropathic pain. We used the well-established spinal nerve ligation (SNL) animal model of neuropathic pain, which mimics clinical symptoms such as mechanical allodynia (pain from non-painful touch) and heat and cold hyperalgesia. We used an integrative discovery-driven approach to generate systems-level hypotheses to be tested in future studies, including the role of neuroimmune signaling, myelination, and specific cell types in the amygdala in the chronification of neuropathic pain.

## 2. Materials and Methods

### 2.1. Animals

Adult male Sprague-Dawley rats (250–400 g, 12 weeks of age at the time of behavioral testing) were group-housed (*n* = 3 per cage) in a temperature-controlled room under a 12 h day/night cycle with ad libitum access to food and water. All experimental procedures were approved by the Institutional Animal Care and Use Committee (IACUC, protocol #21026) of Texas Tech University Health Sciences Center (TTUHSC) and conformed to the guidelines of the International Association for the Study of Pain (IASP) and the National Institutes of Health (NIH).

### 2.2. Neuropathic Pain Model

The well-established SNL rat model of neuropathic pain [21] was utilized to induce a stable and long-lasting peripheral neuropathy. Rats were anesthetized with isoflurane (2–3%; precision vaporizer, Harvard Apparatus, Holliston, MA, USA) and underwent surgery to expose and tightly ligate the left L5 spinal nerve using 6-0 sterile silk. A sham-operated control group underwent a similar surgical procedure, where the L5 spinal nerve was exposed but not ligated. A topical antibiotic (Bacitracin) was applied daily for 5 days after all surgical procedures to prevent infection. To confirm the induction of neuropathic pain, all animals were tested for mechanosensitivity as part of a larger study that used calibrated forceps with a force transducer to measure hindlimb withdrawal threshold [22]. The withdrawal threshold was statistically significantly lower in SNL animals compared to Sham control [22].

### 2.3. RNA Isolation and Bulk Sequencing

At the chronic stage of neuropathic pain (4 weeks post-SNL or post-sham surgery), rats (SNL, *n* = 6; sham, *n* = 6) were euthanized via decapitation. The brains were rapidly extracted and oxygenated in ice-cold artificial cerebrospinal fluid (ACSF) that contained the following (in mM): 125.0 NaCl, 2.6 KCl, 2.5 NaH_2_PO_4_, 1.3 CaCl_2_, 0.9 MgCl_2_, 21.0 NaHCO_3_, and 3.5 glucose. Coronal brain slices (1000 μm) containing the left and right CeA were prepared using a Vibratome (VT1200S, Leica Biosystems, Nussloch, Germany) as described previously [23]. The left and right CeA and BLA were dissected from freshly harvested slices for bulk RNA sequencing analysis. Total RNA was isolated using the MagMAXTM-96 Kit (Life Technologies, Carlsbad, CA, USA) and checked for quality control (all RIN values were >8.4). RNA library preparation and sequencing were performed at the University of Texas at Austin Genomic Facility. Illumina Tag-Seq of polyA-enriched total RNA sequencing was performed (single end, 100 bp). Data were uploaded to GEO with a delayed release date (GSE325961).

### 2.4. Quality Control and Alignment

Read quality was assessed using Fastp (v.0.23.4) [24]. Reads with a Phred score below 30 were removed (-q 30), and 10 low-quality nucleotides were trimmed from the ends of the reads (-t 10), resulting in reads with a mean length of 91 bp. To align the reads to the reference genome, Salmon (v.1.10.2) [25] was utilized. A decoy-aware index was created using the mRatBN7.2 genome release 111 from Ensembl [26]. After building the index, the salmon quant command with the -l A flag was used to automatically identify the library type and the --validatingMappings flag to quantify transcript expression.

### 2.5. Differential Expression Analysis

The transcript expression data for each region (CeA, BLA) was imported into RStudio using tximeta (v1.20.3) [27], and the summarizetogene function was used to create one edgeR object per region. Prior to differential gene expression analysis (DEG), a principal component analysis (PCA) was performed using ggfortify (v0.4.17) to observe the main sources of variation and identify potential outliers. A DEG analysis was then conducted using edgeR (v4.0.16) [28]. Prior to running the QLFtest, the filterbyexpress function was applied to remove low-expressed genes (defined as those with fewer than one count per gene in 40% of the samples). This was followed by the calcnormfactors function to normalize the data. The makecontrast function was used to define the experimental group comparisons. Experimental groups included the following: Sham.Left, Sham.Right, SNL.Left, and SNL.Right. Based on our hypothesis of separate mechanisms in the left and right amygdala underlying neuropathic pain, hemispheric samples were treated as independent observations using a two-factor linear model. For each brain region, genes were identified with the main effect of Pain (SNL vs. Sham), Side (Left vs. Right), and their interaction. Genes showing statistically significant Pain × Side interaction were further analyzed for the effect of Pain within a hemisphere. Genes with a nominal *p*-value < 0.05 were considered DEGs. False discovery rate (FDR) was also calculated and is reported in the Supplementary Tables. Results were visualized using volcano plots and Venn diagrams created with ggplot (v3.5.1), ggvenn (v0.1.10), and ggrepel (v0.9.5) [29]. To generate all heatmaps for DEGs and cell type −log(*p*-value), the package ComplexHeatmap (v2.26.1) with hierarchical clustering using z-scores for all DEGs and the Pearson correlation coefficient as a distance was used. All Pain × Side interaction plots for individual genes were created by ggplot (v3.5.1). A hypergeometric test was used to estimate an overlap between DEGs in BLA and CeA irrespective of direction, with all detected genes common for the two brain regions being used as background.

### 2.6. Functional Group Over-Representation Analysis

Functional group over-representation analysis for biological processes and molecular pathways was conducted using ClusterProfiler (v4.10.1), utilizing Gene Ontology (GO), Reactome, and the Kyoto Encyclopedia of Genes and Genomes (KEGG) databases [30]. DEGs with a nominal *p*-value < 0.05 were used as queries, and all detected genes were used as background. Dot plots representing overrepresented terms were generated with ggplot2 (v3.5.1). To explore connections between overrepresented terms, gene networks were built using Cytoscape (v3.10.3) [31].

### 2.7. Cell Type Over-Representation Analysis

To determine cell type-specific DEGs and cell types most responsive to the factors of Pain, Side, and their interaction, published molecular markers of cell types in the amygdala were used to perform a cellular “deconvolution” and cell type over-representation analyses. Previously published single-nucleus RNA sequencing data [32] (GEO Accession GSE212417) were used to identify cell type-specific genes in the amygdala and to define the cellular identity of DEGs. Seven sample matrices were downloaded from GSE212417, corresponding to the whole amygdala from treatment-naïve rats, and a published protocol [33] and Seurat (v5.1.0) [34] were used to identify cellular clusters and define cell types using canonical cellular markers. Thirteen cell types were defined based on the number of clusters from the dataset: mature oligodendrocytes (OD), immature oligodendrocytes (Immature OD), oligodendrocyte precursor cells (OPC), microglia, astrocytes, endothelial cells, VGLUT1-positive glutamatergic neurons (ExNeuron), VGLUT2-positive, Nos1-positive glutamatergic neurons (Nos1+), intercalated cells (IC Cells), general GABAergic inhibitory neurons (InhNeuron), proenkephalin-positive neurons (Penk+), somatostatin-positive neurons (Sst+), and reelin-positive neurons (Reln+). To identify amygdala cell types over-represented with DEGs, a hypergeometric test (phyper) was run in R with an expression/enrichment filter of 0.6 for the genes from the referenced dataset for each region. A *p*-value < 0.00385 was considered statistically significant after the Bonferroni correction. Up- and down-regulated genes were analyzed separately to explore the directionality of cellular responses to pain. To further investigate the effects of SNL on the expression of all cell type-specific genes of five over-represented cell types (OD, Immature OD, OPC, ExNeuron, and Nos1+) in the CeA and BLA, distribution curves were built based on the t-value statistic for each gene, and mean t-values for each cell type were compared to zero chance using a one-sample *t*-test followed by Bonferroni correction. T-values were calculated from the edgeR-based F statistics (Supplementary Table S1).

### 2.8. Integrative Approach to Data Analysis

Transcriptomics is a multivariate discovery-driven hypothesis-generating approach that has been well-accepted in different fields since the introduction of “omics” two decades ago. Strict control for Type I error results in small lists of genes filtered by statistical significance only, which markedly limits the power of downstream bioinformatics analyses. To balance Type I and II errors and accelerate discovery, we used an integrative systems approach that combines nominal statistical significance for individual DEGs and biological significance at the functional group and cell type levels. At the individual gene level, rigor is achieved by focusing on DEGs that (a) are members of an over-represented functional group/molecular pathway, (b) are markers of a specific cell type, and/or (c) are hubs in their respective molecular networks. We have used this approach to generate focused hypotheses, many of which were validated experimentally [35,36].

## 3. Results

### 3.1. Effects of Chronic Pain (SNL Model) on Gene Expression in BLA and CeA

**Figure 1:**
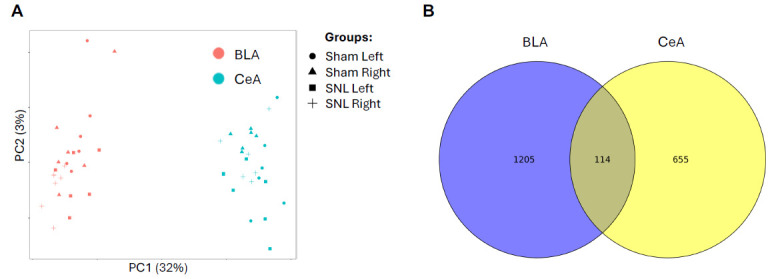
Principal component analysis (PCA) and differentially expressed genes (DEGs). (**A**) PCA separates BLA and CeA samples into different clusters. (**B**) DEGs in BLA and CeA were mainly different; however, the overlap of 114 DEGs was significantly greater than expected by chance (hypergeometric *p*-value < 0.001).

**Figure 2:**
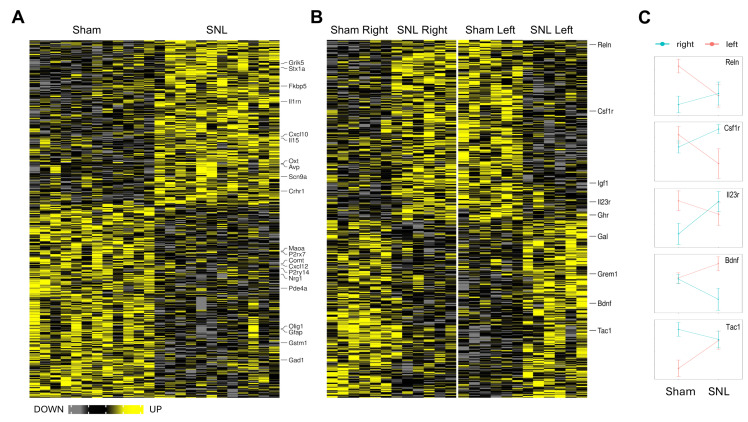
Differentially expressed genes (DEGs) in the CeA. Heatmap plots of individual samples (columns) show all DEGs for the main effect of Pain (**A**) and Pain by Side interaction (**B**). Gene symbols of selected genes with a known or proposed function in pain modulation are shown to the right of each heatmap. Normalized Means ± SEM for selected genes with the Pain × Side interaction effect are shown in (**C**).

**Figure 3:**
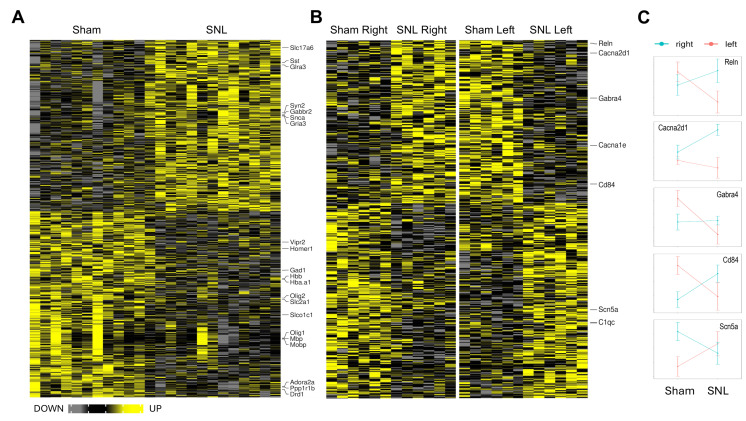
Differentially expressed genes (DEGs) in the BLA. Heatmap plots of individual samples (columns) show all DEGs for the main effect of Pain (**A**) and Pain by Side interaction (**B**). Gene symbols of selected genes with known or proposed function in pain modulation are shown to the right of each heatmap. Normalized Means ± SEM for selected genes with the Pain × Side interaction effect are shown in (**C**).

The well-established SNL animal model of neuropathic pain was used to identify individual genes affected by chronic pain in the CeA (799 DEGs) and BLA (1362 DEGs) (Supplementary Table S1). PCA identified “brain region” as the main source of variability in gene expression, separating the CeA and BLA samples along PC1 axis (Figure 1A), and providing the rationale for separate analyses within each brain region. Lists of DEGs showing the main effect of Pain were largely different between the BLA and CeA (Figure 1B). However, the inter-region overlap of 114 DEGs was significantly greater than expected by chance (hypergeometric *p*-value < 0.001), suggesting some common mechanisms of pain regulation in the two brain regions. These DEGs were further investigated using cell type over-representation analysis (presented below). Heatmaps in the CeA (Figure 2A) and the BLA (Figure 3A) show selected DEGs up- or down-regulated in the SNL group compared to the control. Overall results can be found in Supplementary Table S1.

In the CeA, several identified DEGs were implicated in synaptic transmission (e.g., *Stx1a*, *Crhr1*, and *Oxt*) and neuroimmune signaling (e.g., *Il1rn*, *Cxcl10*, *Cxcl12*, and *P2ry14*), suggesting a potential interplay of these processes in pain regulation. In the BLA, several DEGs were also implicated in synaptic transmission (e.g., *Gria3*, *Drd1*, and *Homer1*), with some genes being molecular markers of specific neuronal types (e.g., *Slc17a6* in glutamatergic neurons, *Gad1* and *Sst* in GABAergic neurons). In addition, several molecular markers of oligodendrocytes implicated in myelination (e.g., *Mbp*, *Plp1*, *Myrf*, and *Olig1*) were down-regulated in the SNL group, suggesting BLA hypomyelination in chronic pain. *Myrf* and *Olig1* were also down-regulated in the CeA.

### 3.2. Hemispheric Lateralization in Pain-Induced Gene Expression

Accumulating evidence suggests right-hemispheric lateralization of pain processing in the amygdala, though relatively little is known about the role of lateralization in chronic pain [18,19,20]. To investigate the potential role of hemispheric lateralization in pain-induced effects on gene expression, DEGs showing Pain × Side interaction were identified, with 613 in BLA and 598 in CeA. Many of such genes showed side-specific regulation by chronic pain, i.e., regulated (either statistically significantly or with a strong tendency) in the opposite directions in the right and left hemispheres four weeks after SNL surgery. A follow-up analysis showed that 12 genes in BLA and 17 in CeA were statistically significantly regulated in opposite directions. Overall results of this analysis are shown in Figure 2B,C and Figure 3B,C for the CeA and the BLA, respectively, as well as in Supplementary Table S1.

In the CeA, several neuronal (e.g., *Reln*, *Bdnf*, and *Tac1*) and neuroimmune (e.g., *Csf1r*, *Il23r*) genes were identified with the interaction effects, which is consistent with the main effect findings. In the BLA, the majority of DEGs were neuronal (*Reln*, *Gabra4*, and *Scn5a*). Figure 2C and Figure 3C show the directionality of SNL-induced changes in the right and left hemispheres.

### 3.3. Functional Group Over-Representation Analysis

**Figure 4:**
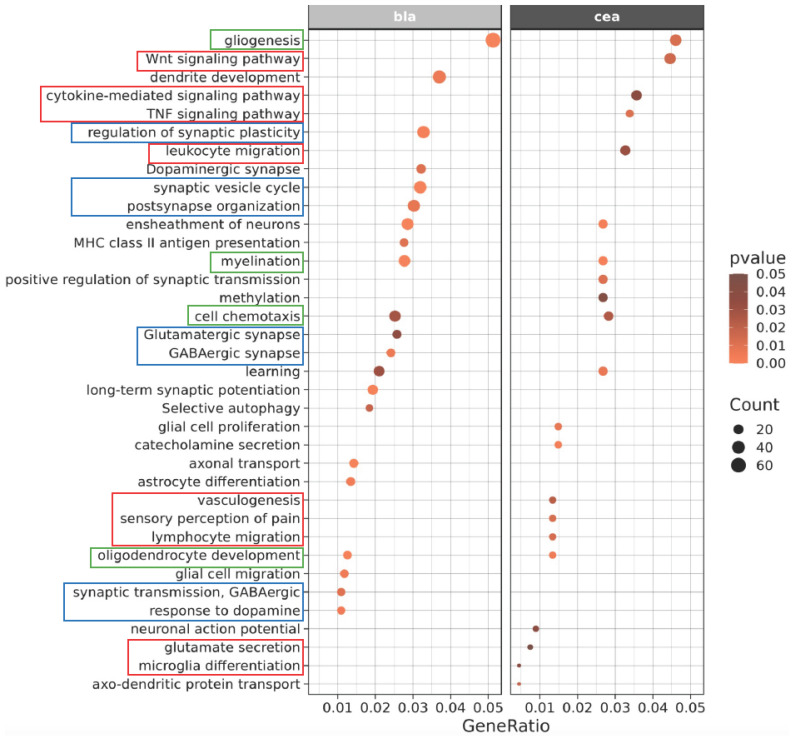
Representative results of functional group over-representation analysis for the effect of Pain (SNL vs Sham) in the BLA and CeA. Green boxes outline selected functional categories that are similarly affected in both brain regions, while red (CeA) and blue (BLA) boxes outline brain region-specific functional groups.

The potential role of different biological categories and molecular pathways was further investigated by performing functional group over-representation analysis for DEG lists. Representative results of this analysis for the effect of Pain (SNL vs Sham) in the CeA and the BLA are shown in Figure 4, and all results can be found in Supplementary Table S2. Over-represented functional groups (OFG) common for the CeA and the BLA included myelination and oligodendrocyte development, both of which mainly contain down-regulated genes. While CeA-specific OFGs tended to include more immune-related categories, such as TNF signaling pathway and cytokine-mediated signaling pathway, the BLA-specific OFGs tended to include more neuron-related categories, such as glutamatergic synapses and regulation of synaptic plasticity.

**Figure 5:**
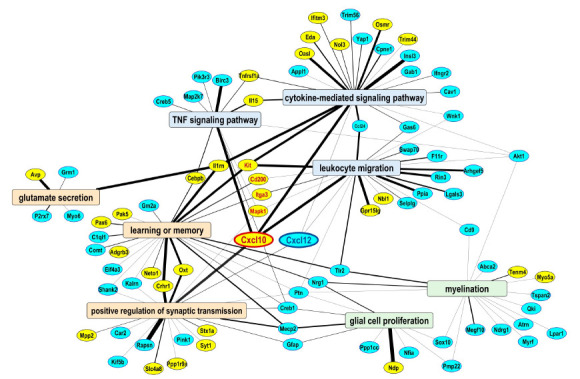
Representative network of over-represented functional groups and associated DEGs in the CeA implicated in the regulation of neuropathic pain. Functional groups from three “domains” are shown: “neuronal” (orange background), “immune” (blue background), and “glial” (green background). DEGs (yellow = up-regulated, blue = down-regulated in SNL vs Sham group) connecting these domains are potential CeA targets for cell–cell interactions in the context of neuropathic pain. Highlighted are two hub genes in the middle, *Cxcl10* and *Cxcl12*, which are chemokines implicated in the communication between microglia, astrocytes, and neurons.

To identify DEGs common for immune-, glia-, and neuron-related processes in the CeA, gene lists of these categories were compared and several genes linking them were determined, including *Cxcl10*, *Cxcl12*, *Il1rn*, and *Cd200* (Figure 5). For example, *Cxcl10* was up-regulated while *Cxcl12* was down-regulated in SNL animals. These genes are chemokines shown to be involved in the communication between neurons and glia, implicating the interplay between these cell types in the regulation of neuroimmune signaling in chronic pain.

### 3.4. Cell Type Over-Representation Analysis

**Figure 6:**
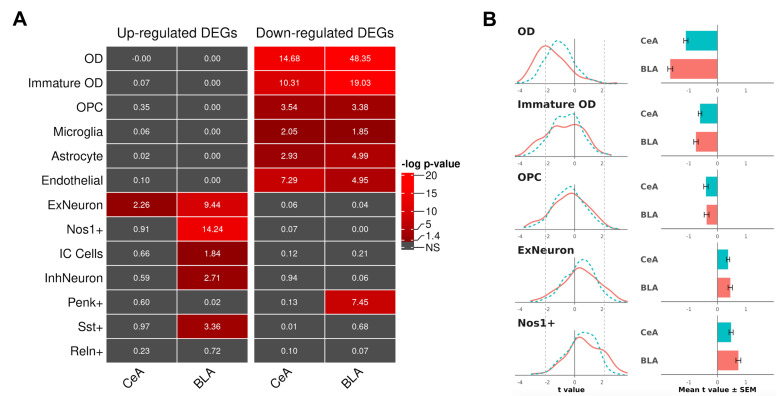
Cell type analysis of genes for the Pain condition (SNL vs Sham) in the BLA and CeA. Publicly available cell type-specific molecular markers were used to predict the cellular identity of all genes with detectable expression in the amygdala. (**A**) Cell type over-representation analysis of up- and down-regulated DEGs. Shown *p*-values are results of hypergeometric tests comparing the numbers of cell type-specific DEGs to chance. A *p*-value < 0.00385 (−log(10) > 2.41) was considered statistically significant after the Bonferroni correction. (**B**) Distributions of t-statistics of all genes enriched in one of five cell types: mature ODs, immature ODs, OPCs, VGLUT1+ glutamatergic neurons (ExNeuron), and VGLUT2+ and Nos1+ glutamatergic neurons. A distribution shift to the left suggests a general down-regulation of cell type-specific genes in SNL vs Sham, while a distribution shift to the right suggests a general up-regulation. Mean t-values for all five cell types (right panels) were significantly different from zero (one-sample *t*-test with Bonferroni correction, all *p*-values < 0.01). Dashed lines next to the −2 and +2 t values indicate thresholds for statistical significance for individual genes. For example, genes with t values <−2 or >+2 are considered DEGs.

To explore the potential role of different cell types in the amygdala in pain regulation, cell type-specific DEGs were identified using published datasets, and cell type over-representation analysis was performed. This analysis revealed two main findings: (1) down-regulated genes in the CeA and BLA were over-represented in non-neuronal cell types, especially in ODs and immature ODs; and (2) up-regulated genes in the BLA were over-represented in neurons, especially glutamatergic neurons such as VGLUT1-positive ExNeuron and VGLUT2-positive Nos1+ (Figure 6A). Distributions of t-statistics for all cell type-specific genes expressed in OD, immature OD, OPC, ExNeuron, and Nos1+ cell types validated these findings, showing overall shifts to the left (down-regulation) or to the right (up-regulation) from zero. Examples of cell type-specific DEGs include OD-specific and down-regulated *Olig1* and *Myrf* in both the CeA and BLA, ExNeuron-specific and up-regulated *Gabbr2* and *Gria3*, and Nos1+-specific and up-regulated *Slc17a6* (VGLUT2) and *Gabrg2* in the BLA. In addition, all OD-specific major myelin-associated genes (*Mbp*, *Plp1*, *Mag*, *Mog*, and *Mobp*) were down-regulated in the BLA, suggesting a loss of myelin in this region in the SNL group. Consistent with functional group and cell type over-representation analyses, the list of 114 DEGs commonly regulated between the CeA and BLA (Figure 1B) was over-represented with OD-specific genes, with all of them being down-regulated (hypergeometric *p*-value < 0.01), suggesting OD dysfunction in both regions as a common mechanism of neuroplastic changes in the amygdala in chronic pain.

## 4. Discussion

This study targeted two regions of the amygdala, the CeA and the BLA, for analysis of neuroplastic changes in chronic pain at the gene expression level using transcriptomic and bioinformatic approaches, and identified individual genes, molecular pathways, biological processes, and specific cell types affected by chronic neuropathic pain in male rats. An integrative approach that focuses on functional significance and cell-type specificity of DEGs was used to nominate mechanistic targets for the central regulation of chronic pain. The present results are consistent with the literature and propose novel avenues for pain research. The analysis of gene expression changes in a chronic pain condition in specific cell types in different amygdala subregions with hemispheric lateralization, using cellular “deconvolution” of transcriptomic data to generate novel cell type-specific hypotheses, significantly advances findings from a previous study at the acute stage of the SNL model [17].

When comparing SNL-induced transcriptomes between the two brain regions, there was little overlap between DEGs in the CeA and BLA. This finding was not surprising given that the two regions have distinct afferent and efferent projections and functional properties [8,10,37,38,39]. The BLA is predominantly composed of pyramidal glutamatergic projection neurons that receive polymodal sensory and nociceptive information from thalamic and cortical areas, including the anterior cingulate cortex (ACC), insula, and medial prefrontal cortex (mPFC) [40,41]. The BLA integrates emotional–affective information and then relays this into the CeA for further processing [6]. The CeA serves as the interface between nociceptive and affective processing and as a major output nucleus that connects to other brain regions involved in behavioral regulation. The BLA-CeA circuit has been implicated in the generation and modulation of pain-like behaviors [42]. Despite largely different effects of pain on the CeA and BLA transcriptomes, the overlap between DEGs in these regions was significantly greater than expected by chance. Many DEGs in this list were highly enriched in OD, with all of them being down-regulated, suggesting OD dysfunction in chronic pain.

Our follow-up cell type-specific analysis revealed a drastic pain-induced decrease in expression of OD-specific genes in the amygdala, particularly within the BLA. Many of the OD-specific DEGs are implicated in the regulation of myelination (e.g., *Mbp*, *Plp1*, *Myrf*, and *Olig1*), suggesting that chronic pain results in general OD dysfunction and demyelination. These findings are somewhat consistent with results from a recent study that explored neural circuits involved in comorbid chronic pain and depression [43]. Their transcriptomic analysis revealed that BLA-ACC hyperactivity was associated with an enrichment for genes expressed by ODs in the ACC; the majority of these were down-regulated and were associated with decreased myelination pathways in this region. Given the central role of ODs in maintaining axonal integrity and metabolic support, the proposed OD dysfunction may have profound functional consequences for local and long-range circuits.

Another main finding in this study is the potential role of neuroimmune signaling in the CeA. Neuroimmune signaling is now recognized as an important peripheral and spinal pain mechanism [44], but the role of neuroimmune factors in pain-related amygdala neuroplasticity and behavior is not well understood. Little is known about the role, regulation, and therapeutic potential of molecular crosstalk between neuronal and glial cell types in the brain in the context of pain. The present results suggest a potential interplay between neuronal and immune processes in pain regulation and nominate several molecular targets as mechanistic candidates for communication between neurons, microglia, and other non-neuronal cell types. For example, two chemokines, *Cxcl10* and *Clcx12*, were found to be up- and down-regulated, respectively, in the SNL group, which connected the “immune” and “synaptic” domains in CeA (Figure 5).

CXCL10 expression is generally low in the healthy CNS but is significantly up-regulated during inflammation, infection, and injury [45]. Microglia and astrocytes are major sources of CXCL10, especially under inflammatory conditions [46]. CXCL10 primarily acts via its receptor, CXCR3, which is expressed on neurons and can modulate synaptic transmission and promote apoptosis of neurons in a dose-dependent manner during chronic inflammation [47]. CXCL10 may be involved in the late phase of neuropathic pain and is up-regulated in astrocytes of the spinal cord after nerve injury or ischemia [48]. CXCL12 is constitutively and widely expressed in the healthy CNS and is crucial for maintaining brain homeostasis [49]. CXCL12 primarily signals through its receptors CXCR4 and, in the adult brain, it modulates neurotransmission, enhances synaptic plasticity, and generally provides neuroprotective effects against various insults [50]. Interestingly, the CXCL12 protein was up-regulated in the dorsal root ganglia and spinal cord one to fourteen days after spared nerve injury in rats (a neuropathic pain model) but returned to the baseline level by day 21 [51]. A down-regulation of *Cxcl12* in this study may indicate a different role of this gene at the chronic stage of neuropathic pain, differences between the CeA compared to the spinal cord, a potential mRNA expression compensation for the up-regulated protein, or all of the above. Taken together, these data suggest a proinflammatory state and disrupted homeostasis in the CeA under chronic pain conditions.

The potential role of hemispheric lateralization in pain-induced effects on gene expression was investigated by identifying DEGs showing Pain × Side interaction. The premise is based on the hypothesis that some genes may be regulated in opposite directions in the right and left hemispheres or only in one hemisphere under pain conditions. Similar to the main effect of Pain, many DEGs with the interaction effect in the CeA were implicated in either neuronal or immune functions (e.g., *Tac1*, *Bdnf*, and *Il23r*). Tachykinin (Tac1) and brain-derived neurotrophic factor (BDNF) are known targets in pain research [52]. For example, Substance P, the peptide encoded by the Tac1 gene, acts as an excitatory neurotransmitter in pain signaling pathways and is widely expressed in pain-related circuitry, including the spino-parabrachio-amygdaloid pain pathway that provides nociceptive information to the CeA [53,54,55]. Also, blocking the signaling of spinal BDNF and its TrkB receptor reversed mechanical allodynia induced by peripheral nerve injury in male rats [56]. Both *Tac1* and *Bdnf* had at least a tendency for pain-induced down-regulation in the right and up-regulation in the left hemispheres, consistent with structural synaptic changes in parabrachial input to the CeA in chronic pain [57]. DEGs with the interaction effects in the BLA were mainly associated with neuronal functions. For example, reelin (*Reln*), a large extracellular matrix protein that plays an important role in brain development and function [58], was down-regulated in the left hemisphere and had a tendency to be up-regulated in the right hemisphere in SNL rats. In the adult brain, reelin is mainly secreted by a subset of GABAergic interneurons including the amygdala [59]; however, the specific role of these neurons in pain conditions has not been directly characterized. The Pain × Side interaction effects were not as robust as the main effects of pain, possibly, due to the relatively limited power of ANOVA to test interactions. Nevertheless, the present results suggest that hemispheric effects are important in the regulation of chronic pain and warrant further investigation.

Cell type over-representation analysis showed that up-regulated DEGs in the BLA were highly over-represented in glutamatergic neurons (both VGLUT1-expressing and VGLUT2-expressing Nos1+ neurons). Many of these DEGs are implicated in the regulation of neuronal excitability and synaptic functions (e.g., VGLUT2), and this up-regulation may indicate an increased excitability of BLA glutamatergic neurons. Nos1+ neurons are a distinct subpopulation of excitatory neurons that may be involved in pain-related anxiety and depression. The BLA Nos1+ and Nos1− neurons have opposite effects on anxiety and depression-like behaviors by projecting to different areas [60]. Together with the evidence for demyelination in this brain region, the present results indicate an imbalance in neurotransmission and point to the BLA as an important player in the regulation of chronic neuropathic pain.

To ensure a rigorous and transparent interpretation of the results, several limitations must be considered. The goal of our exploratory study was to identify molecular and cellular factors associated with pain-related neuroplasticity and formulate testable hypotheses for future studies. Our study is limited to a later stage of chronic pain and only in males. To understand the dynamic process of pain chronification and sex differences, molecular mechanisms should be investigated at multiple time points in both males and females. Our sample size of six per group allowed us to detect moderate changes in gene expression. To evaluate the full spectrum of SNL effects on molecular profiles, larger sample sizes should be considered. Another limitation is the lack of experimental validation. We have a high rate of success for technical validation using qPCR and believe that validating results using this technique is not necessary. Biological (functional) validation will be carried out in future studies. Our robust integrative approach allowed us to formulate testable hypotheses at a systems level. However, interpretation of results at the individual gene level should be taken with caution, especially for DEGs with marginal *p*-values and small fold change. Finally, our cell type-specific analysis is based on previously published cell type-specific molecular markers. Without biological validation, interpretation of cell type-specific findings should be taken with caution.

## 5. Conclusions

In summary, this study provides novel insights into the central regulation of chronic neuropathic pain centered on neuroimmune signaling and myelin changes in the amygdala. Despite some limitations, this research paves the way for future studies examining mechanisms of transition from acute to chronic pain, sex differences, the role of individual cell types, and cell–cell interactions in the right and left amygdala, as well as amygdala-related neurocircuits in the chronification of pain. Overall, these data point to OD dysfunction in the CeA and BLA, neuroimmune signaling in the CeA, and glutamatergic neurotransmission in the BLA as chronic pain mechanisms and potential therapeutic targets for the management of chronic neuropathic pain.

## Data Availability

The datasets generated during and/or analyzed during the current study were uploaded to GEO with a delayed release date (GSE325961).

## Supplementary Materials

The following supporting information can be downloaded at: https://www.mdpi.com/article/10.3390/cells15090775/s1; Table S1: DEGs and cell type enrichment; Table S2: Functional group over-representation analysis.
